# Examining risk factors for cardiovascular disease among food bank members in Vancouver

**DOI:** 10.1016/j.pmedr.2018.04.015

**Published:** 2018-04-23

**Authors:** A.O. Fowokan, J.L. Black, E. Holmes, D. Seto, S.A. Lear

**Affiliations:** a Department of Biomedical Physiology and Kinesiology, Simon Fraser University, Burnaby V5A 1S6, BC, Canada; b Faculty of Land and Food Systems, Food Nutrition and Health, University of British Columbia, 2205 East Mall, Vancouver V6T 1Z4, BC, Canada; c Faculty of Health Sciences, Simon Fraser University, Burnaby V5A 1S6, BC, Canada; d Greater Vancouver Food Bank, Vancouver, BC, Canada; e Division of Cardiology, Providence Health Care, Vancouver V6Z 1Y6, BC, Canada

**Keywords:** Food insecurity, Food banks, Cardiovascular disease, Risk factors, Canada

## Abstract

Food banks provide supplemental food to low-income households, yet little is known about the cardiovascular health of food banks members. This study therefore described cardiovascular disease (CVD) risk factors among food bank members and explored associations between food insecurity and CVD risk.

Adults ≥18 years (n = 77) from three food bank sites in metro Vancouver, British Columbia completed surveys and physical assessments examining a range of socio-demographic variables and CVD risk factors. A composite measure of myocardial infarction (MI) risk called the INTERHEART score was assessed and household food insecurity was measured using the Household Food Security Survey Module. Regression models were used to explore associations between food insecurity and CVD risk measures, including the INTERHEART score.

Ninety-seven percent of food bank members reported experiencing food insecurity, 65% were current smokers, 53% reported either chronic or several periods of stress in the past year, 55% reported low physical activity levels and 80% reported consuming fewer than five servings of fruit and vegetables daily. Prevalence of self-reported diabetes and hypertension were 13% and 29% respectively. Fifty-two percent of the sample were at high risk of non-fatal MI. No statistically significant associations were found between increased severity of food insecurity and CVD risk factors among this sample where both severe food insecurity and high CVD risks were prevalent.

Food bank members were at elevated risk for CVD compared with the general population. Strategies are needed to reduce prevalence of food insecurity and CVD risk factors, both of which disproportionately affected food bank members.

## 1 Introduction

Food insecurity, defined as the “*lack of secure access to sufficient amounts of safe and nutritious food for normal growth and development and an active and healthy life*” (FAO, 2000, p, 26), is a public health concern (Rideout and Kosatsky, 2014; Surgeon General, 2016) and an important social determinant of health (Wilkinson and Marmot, 2003). A prominent response to food insecurity in North America has been through charitable organizations including food banks, which distribute supplemental food to individuals in need (Riches, 1997).

Evidence from the United Kingdom, United States and Canada suggests that food bank use has grown drastically in the past three decades (Feeding America, 2014; Food Banks Canada, 2016; Loopstra et al., 2015). For example, the number of food banks in Canada rapidly proliferated since first established in 1981 to become a common service providing emergency food to an estimated 860,000 people per month in 2016 (Riches, 1997; Food Banks Canada, 2016). And while some food banks now incorporate health promotion services (Wakefield et al., 2013), little is known about the health challenges of food bank members, as they remain underrepresented in research studies. The few studies that do exist have focussed on nutritional quality of foods offered and clients' needs, but have overlooked chronic disease risk factors or their connections with food insecurity (Bazerghi et al., 2016; Simmet et al., 2017). Given that food insecurity is associated with adverse health outcomes, including chronic diseases, it is important to characterize the health risks of food bank users. Therefore, this study describes risk for CVD, and explores the associations of food insecurity with CVD risk factors among food bank members in metro Vancouver.

## 2 Methods

### 2.1 Study population

Participants were recruited from the Greater Vancouver Food Bank (GVFB). The GVFB offers supplemental food at no cost to approximately 6500 individuals weekly across 13 community sites in metro Vancouver, Canada. Three of these sites were selected in conjunction with the GVFB for recruitment as they serve diverse neighborhoods and members.

Food bank members (n = 77) age 18 years and older, with the ability to communicate in English, were recruited using convenience sampling. Data collection was carried out by trained research assistants in a private space at the food bank sites and assessed food insecurity status, CVD risk factors, demographic and socio-economic variables. All participants provided informed consent. This study was approved by the Research Ethics Boards from Simon Fraser University and the University of British Columbia.

### 2.2 Demographic and socio-economic status assessment

Socio-economic status was assessed through annual income level and government income assistance use (social assistance and/or disability benefits) using questions formulated in consultation with the GVFB. Income was categorized to reflect incomes below 2015 income assistance rates and above measures that correspond to the Canadian low-income cut-offs (Statistics Canada, 2016). Demographic variables such as ethnicity and education were determined using questions from the Canadian Community Health Survey (CCHS), while questions on household size and household structure were adapted from the National Household Survey (Statistics Canada, 2011; Statistics Canada, 2015).

### 2.3 Household food insecurity assessment

Food insecurity status was determined using the validated Household Food Security Survey Module (HFSSM) (Health Canada, 2004) which assesses household food behaviours in the previous 12 months including access to food and the inability to meet the required food needs due to limited financial resources (Health Canada, 2004). The questionnaire combines a 10-item Adult Food Security Scale and an 8-item Child Food Security Scale that probes experiences of children. For households with children, scoring was based on the group (i.e. adult or children) with the most severe level of food insecurity. Because no child scores were categorized higher than adult scores in this study, this analysis draws on the 10-item Adult Food Security Scale score only. For this analysis, food insecurity categorization followed the method outlined by the PROOF research group in Canada, which recognizes four classifications of food insecurity: a) food secure (zero affirmed questions on the HFSSM); b) marginally food insecure (one affirmed question); c) moderately food insecure (two to five affirmed questions); d) severely food insecure (six or more affirmed questions) (Tarasuk et al., 2014).

### 2.4 Cardiovascular disease risk factors and self-reported health status

Cardiovascular risk factors were determined using questions developed for the INTERHEART score (McGorrian et al., 2011). Questions included participants' self-reported smoking status, exposure to second hand smoke, diagnosis of diabetes or hypertension, and their biological parents' history of heart attack. Physical activity was self-reported and categorized as sedentary, mild, moderate or vigorous depending on the level and intensity of activity. Dietary factors were self-reported and included questions regarding consumption frequency of salty foods, deep-fried or fast foods, meat or poultry, and fruit and vegetables over the last month. Consumption of fruits and vegetable was considered protective, while the other dietary behaviours were not. Self-reported life stress experienced in the past year was categorized as no stress, some periods of stress, several periods of stress, or permanent stress.

Waist circumference (WC) and hip circumference (HC) were measured using standardized methods (WHO, 2008). Abdominal obesity was defined using WC >102 cm for men and WC > 88 cm for women. Waist-to-hip ratio was calculated as waist over hip circumference. Blood pressure was assessed using an electronic BP monitor (OMRON, 5 series Model BP-742). Two measures were taken from the left arm over a 5-minute period and the average was recorded.

### 2.5 Cardiovascular disease risk score

Overall risk for CVD was determined using the non-laboratory based INTERHEART score; an aggregate risk score that provides an estimate of an individual's risk of developing a myocardial infarction (MI) in the next 3.25 years (McGorrian et al., 2011). The non-laboratory INTERHEART score is particularly useful in settings where more invasive data collection (e.g. blood draws to determine lipids levels) is challenging. The INTERHEART risk score includes the following: age, sex, diabetes, hypertension, family history of heart disease, smoking status, exposure to second hand smoke, stress, physical activity, dietary factors and waist to hip ratio. Participants are then categorized as low (0–9 points), moderate (10–15 points) and high risk (16–48 points) for non-fatal MI based on their INTERHEART scores. A one-point increase in INTERHEART score was associated with a 14% increased odds of a non-fatal MI in an internationally validated cohort (McGorrian et al., 2011).

### 2.6 Statistical analyses

Descriptive statistics for continuous variables were reported as means (standard deviation), and as counts (percentages) for categorical variables. Sex differences in the continuous variables were assessed using an independent *t*-test, while associations between categorical variables were assessed using Chi-square tests.

Linear regression models (adjusted for age, sex, household income) were used to model associations between the food insecurity severity categories and CVD risk outcomes (BP, WC, waist-to-hip ratio, and the INTERHEART score) as dependent variables. Statistical analysis was done using SPSS v. 22.0 and statistical significance was set at p < 0.05.

## 3 Results

Forty-six males and 31 females between 25 and 83 years of age participated (Table 1). Fifty one percent of participants reported length of food bank usage between one and five years. Participants were mostly unemployed (81%), received social assistance (84%), and lived in adult only households (90%). Most participants (68%) reported a household income below $14,400 per year. Two-thirds (66%) of the participants were severely food insecure indicating reduced food intake and disrupted eating patterns, while 30% were moderately food insecure, suggesting that food quantity and/or quality had been compromised.

**Table 1:** Socio-demographic and health characteristics of food bank members (n = 77).

Characteristics	n (%)
Sex	
Male	46 (60)
Age (years)	
18–40	13 (17)
41–64	51 (67)
≥65	12 (16)
Food bank length of use	
<6 months	8 (10)
6–11 months	4 (5)
1–5 years	39 (51)
>5 years	25 (33)
Food insecurity status^a^	
Food secure	2 (3)
Marginal	1 (1)
Moderate	22 (30)
Severe	49 (66)
Educational status	
<High school	30 (39)
High school graduate	13 (17)
Some post-secondary education	25 (33)
University degree or diploma	7 (9)
Annual household income	
$0–7199	9 (12)
$7200–14,399	43 (56)
$14,400–21,599	17 (22)
$21,600–$28,799	2 (3)
>$28,800	5 (7)
Household structure	
Adult only	69 (90)
2 Adult households w/ children <18	6 (8)
Single parent households	2 (3)
Household size	
1	45 (58)
2	15 (20)
≥3	17 (22)
Employment status	
Unemployed	62 (81)
Government income support^b^	
Social assistance	65 (84)
Disability benefits	47 (61)
Ethnicity	
White/Caucasian	35 (47)
First Nations/Metis/Inuk	18 (24)
Canadian - unspecified	8 (11)
Other	14 (19)
Smoking status^c^	
Never	15 (20)
Former	11 (14)
Current	50 (65)
Perceived life stress in the past year	
Never	11 (14)
Some periods of stress	24 (31)
Several periods of stress	30 (39)
Permanent or chronic stress	11 (14)
Physical activity^d^	
Sedentary	9 (12)
Mild	33 (43)
Moderate	26 (34)
Vigorous	8 (10)
Diabetes^e^, ^d^	
Yes	10 (13.0)
Hypertension^e^, ^d^	
Yes	22 (29)
Parent's history of heart disease	
Yes	29 (38)
No	38 (49)
Unsure	9 (12)
Abdominal obesity^f^, ^e^	
Total	30 (39)
Men	12 (16)
Women	18 (23)
Salty food intake (daily)	
<1 time	54 (71)
1–2 times	18 (24)
>2 times	4 (5)
Deep fried and fast foods (weekly)	
<1 time	57 (75)
1–2 times	14 (18)
>2 times	5 (7)
Fruit and vegetable intake (daily)	
<1 serving	19 (25)
1–4.9 servings	42 (55)
≥5 servings	15 (20)
Meat and poultry (daily)	
<1 time	69 (91)
1–2 times	7 (9)
>2 times	0 (0)
INTERHEART	
Low (0–9 points)	8 (10)
Medium (10–15 points)	23 (30)
High (16–48 points)	40 (52)

a Categorizations of food insecurity in this table are based off the adult scores — Food secure: zero affirmed questions on the Household Food Security Survey Module (HFSSM); Marginal food insecurity: participants who affirmed one question on the adult scale; Moderate food insecurity: participants who affirmed two to five questions on the adult scale; Severe food insecurity: participants who affirmed six or more questions on the adult scale.

b Government social support programs include: BC Employment & Assistance (BCEA), Old Age Security (OAS), Guaranteed Income Supplement (GIS), Child Benefits (Child Tax Benefit, BC Child Benefit, Universal Childcare Benefit), Rental Housing Assistance (Rental assistance program, Shelter Aid). Disability benefits include: BCEA for Person with Disabilities (PWD), BCEA Persons with Persistent Multiple Barriers (PPMB).

c Categorized as never, former (not smoking ≥12 months) or current smoker.

d Sedentary: not physically active; Mild activity: activity requiring minimal effort (e.g. light walking, stretching, yoga); Moderate exercise (e.g. brisk walking, cycling); Vigorous activity: activity that requires large effort and results in rapid heartbeat (e.g. running, sports, swimming etc.)

e Diabetes and hypertension were assessed by asking the participants the following questions: “Do you have diabetes?” and “do you have hypertension?”.

f Abdominal obesity = Waist circumference > 102 cm for men, and >88 cm for women.

The majority of participants were current smokers (65%) and reported either chronic stress or several periods of stress in the past year (53%) (Table 1). Twelve percent of the sample reported no physical activity, while 43% reported mild levels of physical activity. Self-reported prevalence of diabetes was 13%, while hypertension was 29%. The prevalence of measured abdominal obesity was 39%. Fifty two percent of the sample was found to be at high risk of a non-fatal MI based on their INTERHEART scores.

There were no significant differences between men and women for systolic BP, diastolic BP, WC, HC, smoking status, diabetes, hypertension, and INTERHEART (p > 0.05 for all) (Table 2). However, mean waist to hip ratio (p = 0.04) was significantly higher among men. Women were on average at moderate risk and men at a high risk of developing a MI over 3.25 years.

**Table 2:** CVD risk profile of study participant stratified by sex.

Variable	Men (n = 45)	Women (n = 31)	p value for comparison
Age (years)	51.0 ± 13.0	53.9 ± 10.9	0.31
Systolic BP (mm Hg)	134 ± 20	127 ± 16	0.09
Diastolic BP (mm Hg)	84 ± 11	83 ± 10	0.58
Waist circumference (cm)	94.6 ± 12.3	94.6 ± 15.0	0.99
Waist to hip ratio	0.93 ± 0.1	0.90 ± 0.1	0.04
INTERHEART risk score	18.0 ± 6.3	15.5 ± 6.0	0.11
Smoking status (current smokers)	30 (67%)	20 (65%)	0.85
Diabetes	4 (9%)	6 (19%)	0.19
Hypertension	14 (33%)	8 (26%)	0.53

T-test and Chi-square test were used to explore differences in continuous and categorical variables respectively.

Variables presented as counts (%) for categorical variables or mean ± standard deviation for continuous variables. BP = Blood Pressure.

INTERHEART score categories: low risk (0–9), moderate risk (10–15) and high risk (16–48).

One participant declined to be assessed for physical and health measurements.

No significant associations were detected between food insecurity severity with BP, WC, waist-to-hip ratio and the INTERHEART risk score in adjusted or unadjusted models.

## 4 Discussion

In this exploratory investigation of food bank members, the prevalence and severity of both food insecurity and CVD risk factors was high. These findings add insight into a hard-to-reach study population and align with previous evidence that have demonstrated increased cardiovascular disease (CVD) risk factors including obesity, diabetes, and high blood pressure (BP) in food bank members (Robaina and Martin, 2013).

Compared to published data of the general population in British Columbia, participants in our study had a higher prevalence of CVD risk factors including hypertension (29% vs 16%), diabetes (13% vs 6%), current smokers (65% vs 14%), and life stress (53% vs 24%) (Statistics Canada, 2014). A US study also reported a high prevalence of CVD risk factors in users of food banks with hypertension at 68%, diabetes at 26%, and obesity near 40% (Robaina and Martin, 2013). These comparisons highlight the stark disproportionate CVD risk burden experienced by food bank members.

Previous work has reported significant associations between food insecurity and CVD risk using the 10-year Framingham score in a nationally representative US study where both the prevalence and severity of food insecurity were far lower than our current sample (Ford, 2013). In contrast to Ford (2013), we found no association between food insecurity and the INTERHEART risk score. It is likely that the small sample size and lack of variation in food insecurity status in this population reduced the power to detect significant associations. However, the mean INTERHEART score, which places these food bank members at an increased risk of a non-fatal MI in 3.25 years, provides insight into the potential CVD risk faced by many food bank members. As CVD is a multifactorial disease, the value of a risk score is its ability to identify individuals at high risk when the individual risk factors themselves may not necessarily be high.

Lastly, it is also worth noting that the increased presence of CVD risk factors and outcomes we found in this study could likely exacerbate the severity of food insecurity or confine individuals to chronic food insecurity (Tarasuk et al., 2013). The mechanisms that might result in the aforementioned link are as follows: the presence of CVD risk factors could place constraints to individual finances, or the inability to participate in the labour market, thus increasing the likelihood of vulnerability to food insecurity (Tarasuk et al., 2013). This understanding has important implications for policy makers and service providers in addressing the costs of ill health and food insecurity (Tarasuk et al., 2015).

Notable study limitations include the small sample size and lack of variation in food insecurity outcomes which reduce the power to detect significant associations with CVD outcomes. This sample overrepresented longer-term, English speaking members and single person households; in addition, data are needed from other regions to confirm findings beyond metro Vancouver. The non-laboratory based INTERHEART score was designed for use in field settings where obtaining a blood sample is challenging. Although it does not consider blood lipid values, it was comparable in a validation study with INTERHEART scores that included laboratory-based values (McGorrian et al., 2011). Strengths of this study are in the extensive nature of the socio-demographic and CVD variables examined.

Overall, findings here show that a sample of predominantly long-term food bank members were at elevated risk for CVD based on the high prevalence of risk factors and elevated INTERHEART risk score. Given these findings, improved strategies are needed to reduce barriers to food security and increase access to health promotion and disease prevention programs among food bank members.

## Funding/conflicts of interest

This project was funded by the Canadian Institutes of Health Research (FRN: 139544). The GVFB additionally received funds from the McConnell Foundation to support staff time contributing to this project. Authors have no conflicts of interest to disclose.
